# Feasibility of Using Shear Wave Ultrasonic Probes as Pump-Wave Sources in Concrete Microcrack Detection and Monitoring by Nonlinear Ultrasonic Coda Wave Interferometry

**DOI:** 10.3390/s22062105

**Published:** 2022-03-09

**Authors:** Belfor A. Galaz Donoso, Siva Avudaiappan, Erick I. Saavedra Flores

**Affiliations:** 1 Departamento de Física, Universidad de Santiago de Chile, Av. Víctor Jara 3493, Estación Central, Santiago 9170020, Chile; 2 Civil Engineering Department, University of Santiago of Chile, Av. Víctor Jara 3659, Santiago 9170201, Chile; siva.avudaiappan@usach.cl (S.A.); erick.saavedra@usach.cl (E.I.S.F.)

**Keywords:** nonlinear coda wave interferometry, damage detection, concrete, microcracks

## Abstract

This paper represents a first attempt to study the feasibility of using shear wave (SW) ultrasonic probes as pump-wave sources in concrete microcrack detection and monitoring by Nonlinear Ultrasonic Coda Wave Interferometry (NCWI). The premise behind our study is that the nonlinear elastic hysteretic behavior at microcracks may depend on their orientation with respect to the stationary wave-field induced by the pump-wave source. In this context, the use of a SW probe as a pump-wave source may induce the nonlinear elastic behavior of microcracks oriented in directions not typically detected by a conventional longitudinal pump-wave source. To date, this premise is hard to address by current experimental and numerical methods, however, the feasibility of using SW probes as a pump-wave source can be experimentally tested. This idea is the main focus of the present work. Under laboratory conditions, we exploit the high sensitivity of the CWI technique to capture the transient weakening behaviour induced by the SW pump-wave source in concrete samples subjected to loading and unloading cycles. Our results show that after reaching a load level of 40% of the ultimate stress, the material weakening increases as a consequence of microcrack proliferation, which is consistent with previous studies. Despite the lack of exhaustive experimental studies, we believe that our work is the first step in the formulation of strategies that involve an appropriate selection and placement of pump-wave sources to improve the NCWI technique. These improvements may be relevant to convert the NCWI technique into a more suitable non-destructive testing technique for the inspection of microcracking evolution in concrete structures and the assessment of their structural integrity.

## 1. Introduction

The CWI technique was first introduced by Snieder [1] and consists of using the high sensitivity of multiple scattered waves (over 100 kHz for concrete) to capture small disturbances occurred during their propagation time. Such disturbances may be induced by variations in the sound speed, relative changes in the position of the scatters, and/or position of the sources [2,3,4], among others. In structural concrete, several studies have shown that the CWI technique can be used to monitor sound speed changes produced by stress variations [5,6,7,8,9,10,11,12]. The high sensitivity of the CWI technique is a consequence of the large travel paths of the multiple scattered waves, which also make the estimated sound speed variation a global parameter of the material. Other studies have shown that the mixing of the CWI technique with a low-frequency sweep of high amplitude waves (pump-wave source), makes it possible to estimate the small transient reduction of sound speed (weakening behavior) induced by the apparition of a nonlinear elastic hysteretic behavior at microcracks [13,14,15]. This behavior arises from different nonlinear interaction mechanisms present between microcracks surfaces as a consequence of the displacement field produced by the stationary pump-wave. In this context, the use of a frequency sweep and the auto-amplification behavior of stationary waves increase the chance of mobilizing more microcracks. The gradual microcrack proliferation as a product of external cyclic loading produces a transient weakening of concrete material, which can be monitored by the CWI technique. The combination of CWI with pump waves (nonlinear modulator) is referred to as the NCWI technique.

While extensive research on the optimisation of the CWI technique has been performed (for example, thermal bias control technique [8,9,10], small damage localisation [11], selection of frequency range [12], use of continuous wave excitation [16], use of embedded ultrasonic sensors to apply the CWI technique to concrete structures under operational conditions [17,18,19,20], and simulation studies by numerical approaches as the discrete element method [21]), only limited studies on the optimization of nonlinear modulation processes have been performed to date [22,23,24]. These studies have been focused mainly on the influence of the pump-wave emitter position, temperature variation and combining the NCWI technique with the well-know time-reversal method. Recently, simulation studies using the 2D spectral element method address the problem of the quantitative relation between the NCWI measurements and microcrack parameters such as crack length and density [25,26,27,28,29]. These studies are consistent with previous experimental results, but the numerical methodology is unable to simulate directly the nonlinear elastic hysteretic behavior produced by the pump-wave source. To the best of our knowledge, the use of SW probes have been tested only with conventional nonlinear acoustic techniques such as Nonlinear Resonance Ultrasound Spectroscopy (NRUS) [30], but not with the NCWI technique. In this context, this work aims to study the feasibility of using an SW probe emitter as a pump-wave source in the NCWI technique for concrete microcracks detection and monitoring under laboratory conditions. The premise behind our study is that the nonlinear elastic hysteretic behavior at microcracks may depend on their relative orientation with respect to the stationary wave-field induced by the pump-wave source. We believe that the use of an SW pump-wave source as a nonlinear modulator may show some advantages over the use of a longitudinal pump-wave source alone. If we consider that after the initiation process microcracks are randomly oriented in both transverse and longitudinal directions, then a SW pump-wave source can be used to induce the nonlinear elastic hysteretic behavior of microcraks oriented along directions that may not be probably mobilized by a longitudinal pump-wave source placed a the same position. This could be particularly relevant after loading levels that induce microcrack growth, coalescence, and further propagation along preferential directions that depend on the loading direction [31] and if we consider the practical limitations for the placement of ultrasonic probes in concrete structures. Thus, the use of SW pump-wave sources in conjunction with longitudinal pump-wave sources and strategic placement on the concrete structure may help to improve the performance of the NCWI technique. These improvements may be relevant for the inspection of microcracking evolution in concrete structures under operational conditions and for the assessment of their structural integrity, where the structure size and a specific loading condition can limit the performance of the NCWI technique. Here, a strategic selection (between longitudinal, shear-wave or dual-mode ultrasonic probes) and placement of pump-wave sources may be used during the embedding process of ultrasonic sensors within the concrete as the CWI studies described before [18,19,20].

This paper is organized as follows. Section 2 presents the experimental plan and a general description of the NCWI technique. Within this section; we first describe the concrete sample preparation in Section 2.1, the description of the CWI technique in Section 2.2, the theoretical background of the nonlinear elastic hysteretic behavior and the implementation of the NRUS technique in Section 2.3, and the implementation of the NCWI technique in Section 2.4. Section 3 shows the results of the conventional nonlinear resonance ultrasound spectroscopy (NRUS) technique using SW probes and the application of the NCWI technique in concrete samples subjected to different levels of cyclic loading. The main discussions of the present work are presented in Section 4. Finally, our conclusions and recommendations are outlined in Section 5.

## 2. Materials and Methods

The NCWI technique has already been implemented in the context of granular media using consolidated glass bead samples as analogue models of the fault gouge for the study of stick-slip phenomena present in earthquakes and consolidated granular media [32,33]. Basically, it consists of using the high sensitivity of the CWI technique to capture small transient changes in the sound speed induced by a pump-wave source. A stationary ultrasonic wave field of high amplitude induces displacements at the microcrack level. Due to the interacting mechanisms occurring between microcrack surfaces, displacements produce a nonlinear mechanical hysteresis response that eventually leads to a transient material weakening behavior, that is, a transient sound speed reduction.

**Figure 1:**
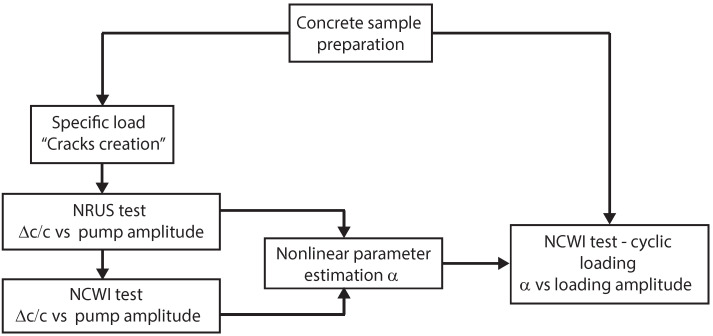
Experimental plan. A cubic concrete sample is submitted to a specific load to induce microcracking and then it is tested by the NRUS technique. This test is used to implement the NCWI method. Once the device settings and processing signal parameters are selected, the NCWI setup is used to test a new concrete sample as a function of the compression loading amplitude.

In this section, we describe the concrete sample preparation, then we present a brief description of the CWI technique and its implementation. The physical principles behind the nonlinear elastic behavior in conjunction with the NRUS method are also explained along with the implementation of the NCWI technique. Our experimental plan consists of testing a concrete sample with the NRUS technique and using this as a basis for the implementation of the NCWI technique. After this preliminary study, we perform the NCWI tests at different levels of compression loads (Figure 1).

### 2.1. Preparation of Concrete Specimens

Ordinary Portland Cement 53 grade of concrete was used for the present investigation. Fine and coarse aggregates were specified according to IS 383-2009 standard [34]. For 1 $m^{3}$ of concrete, the mix consisted of 380 kg of cement, 711 kg of fine aggregate, 1283 kg of coarse aggregate, and 160 L of water, with a water/cement ratio of 0.42. The range of coarse aggregate size was 12.5–20 mm and fine aggregate retaining at 4.75 μm sieve. Both the coarse and fine aggregates were mixed and cement was added to the mixer. These ingredients were mixed at least for 30 s in a dry state and the water was added slowly, which was mixed at least 5 min/max until concrete flowed. The workability was checked by the initial slump to confirm the M30 grade of concrete with water/cement ratio 0.42 attained. We note that if the concrete is mixed beyond the maximum time it could reduce its slump due to the heat of hydration caused during the mixing process. A slump of 125–150 mm was adopted for the present investigation. Once the slump and workability accomplished the standards, concrete cubes were cast with three different layers and each layer consisted of 50 mm thick and 35–45 blows for better compaction. The cast cubes were placed in a room temperature, on a vibration-free sturdy platform to avoid cracks during the shifting of cubes. To reduce the water evaporation in the cubes, a plastic cover was placed on top of the cubes for 24 h. Finally the cast concrete cubes were de-moulded after 24 h and placed in water for curing at room temperature for 3, 7, 14 and 28 days.

### 2.2. Coda Wave Interferometry (CWI)

**Figure 2:**
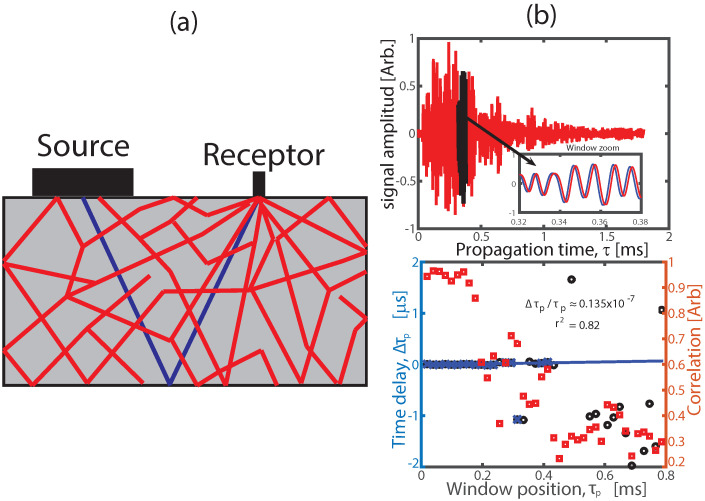
CWI technique. (**a**) Schematic diagram showing the principle of the CWI technique with a coda wave source and a small receptor. The coherent pulse is shown in blue while the diffuse waves are shown in red. (**b**) Sound speed estimation by fitting the filtered curve $\Delta \tau_{p}$ against $\tau_{p}$ (blue). In Figure 2b above, two consecutive coda signals are shown and in the inset it is highlighted the time-shift of a specific window (black). In Figure 2b below it is shown the $\Delta \tau_{p}$ against $\tau_{p}$ (blue and black) and $CC(\tau_{p})$ (red), which is used as a filter to select the good estimates (blue).

The CWI technique consists of using heterogeneous media like an interferometer, which takes advantage of its dispersive characteristics to amplify any small change in sound speed. To do this, we need to transmit ultrasonic waves in the multiple scattering regimen (over 100 kHz for concrete) and capture these signals with an ultrasonic probe of a small area (Figure 2a). The multiple scattered signals are captured repetitively during the evolution time where the concrete sample is subjected to external disturbances as cyclic loading. The CWI signal processing consists of dividing the signals in several identical windows with central position $\tau_{p}$ (*p*: window index) and computing the time-shift $\Delta \tau_{p}$ between coda signals captured at different evolution times by using the cross-correlation function, Equation (1):

(1) $$CC\left(\tau_{p}\right)=\frac{\int x_{pre}\left(t-\Delta \tau_{p}\right)x_{post}\left(t\right)dt}{\int x_{pre}^{2}\left(t-\Delta \tau_{p}\right)dt\int x_{post}^{2}\left(t\right)dt}\;.$$

If there is any perturbance between the coda signals $x_{pre}\left(t\right)$ and $x_{post}\left(t\right)$ the time-shift $\Delta \tau_{p}$ will maximise Equation (1). This computation is performed for the entire coda signal and the slope of $\Delta \tau_{p}$ against $\tau_{p}$ gives directly the sound speed variation $\Delta c/c_{0}$ and the correlation values $CC(\tau_{p})$ are used as quality parameter to filter bad time-shift estimated values (see Figure 2b).

### 2.3. Nonlinear Hysteretic Behavior and NRUS Technique

Heterogeneous materials as concrete exhibit strong elastic nonlinearity due to different physical reasons, including opening/closing microcracks or frictional behavior, among others. As a result, these materials exhibit a hysteresis behavior in their stress-strain response, with slow recovery dynamics and memory effect. Based on Presisach-Mayergoyz space description [30], a nonlinear and hysteretic modulus, without slow dynamics behavior can be written in one dimension. That is:

(2) $$M\left(\varepsilon ,\dot{\varepsilon }\right)=M_{0}(1-\beta \varepsilon -\delta \varepsilon^{2}-\;\cdot \;\;\cdot \;\;\cdot \;-\alpha \left(\Delta \varepsilon +sign\left(\dot{\varepsilon }\right)\varepsilon \right)\;,$$

where $M_{0}$ is the linear modulus, ε is strain, $\dot{\varepsilon }$ the strain rate, β and δ the second and third order non-linear parameters, α the nonlinear hysteretic parameter, and Δε the driven amplitude. Depending on the sign of β, δ and α, this model describes the hardening and weakening of heterogeneous materials with the increment of the driving amplitude of standing waves used in the NRUS technique. In materials such as concrete, at large driving amplitude levels, empirical evidence [30] suggests that the nonlinear elastic hysteretic behavior dominates and the factor α is negative. It means if a material is weakening, it produces a resonant frequency shift proportional to the driven amplitude:

(3) $$\frac{f-f_{0}}{f_{0}}=\frac{c-c_{0}}{c_{0}}\approx \alpha \Delta \varepsilon \;.$$

**Figure 3:**
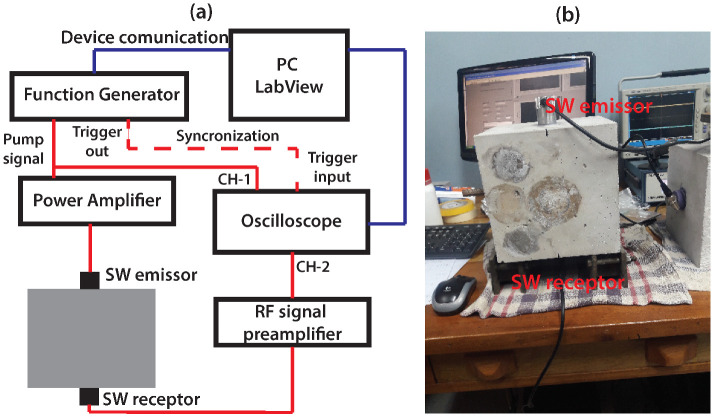
Nonlinear Resonance Ultrasonic Spectroscopy technique. (**a**) Experimental NRUS devices configuration schema. (**b**) Image of the NRUS experiment with the cubic concrete sample of L=20 cm.

Equation (3) is the basis of the nonlinear modulation of the NCWI technique. Before performing any study of the NCWI technique we first implemented the NRUS technique using two identical SW ultrasonic probes placed on a cubic concrete sample of L=20 cm, previously subjected to a specific load to corroborate the possibility of inducing nonlinear elastic behavior with our SW ultrasonic probes (Figure 3). To assure a solid matching between the SW probes and the concrete sample we used phenyl salicylate as a couplant. The shear wave speed, $c_{s}=2641$ m/s, was first measured by the pulse transmission method in order to select a mode, $L=n\lambda \Rightarrow f=nc_{s}/L=52.82$ kHz, within the bandwidth of the SW probes (ultrasonic SW transducer of central frequency 50 kHz, CTS Valpey-Fisher Corporation, model CSSO104GP) and then the frequency shift was measured as a function of the driven amplitude. To achieve this, a function generator (WaveTek, model 80) was controlled through LabView software to produce a linear sweep of 52.4 to 53.4 kHz (resonant mode n=4) with an incremental driven amplitude from 10 mV to 1 V connected to a power amplifier of gain factor 50. The transmitted signals were amplified by RF-Signal Preamplifier (Stanford Research Systems, model SR44S) and were captured in conjunction with the emitted signals by a digital oscilloscope (Tektronix DPO 4034) also controlled through LabView software. Both signals were then stored in a PC for posterior signal processing.

### 2.4. Experimental NCWI Setup

**Figure 4:**
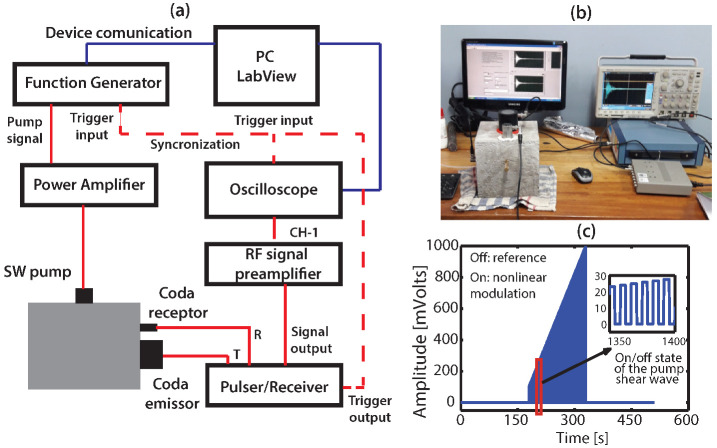
Nonlinear Coda Wave Interferometry technique. (**a**) Experimental NCWI devices configuration schema. (**b**) Image of the NCWI experiment with the cubic concrete sample for device settings performance (same concrete sample than Figure 3). (**c**) SW pump-wave amplitude ramp.

We implemented the NCWI technique to monitor the microcrack proliferation in concrete samples with a similar methodology described in [13], but using an SW ultrasonic probe as a nonlinear modulator (SW pump-wave source). As shown in Figure 4, a coda transmitter sends (ultrasonic transducer of central frequency 250 kHz, Panametrics, model V1012 Videoscan) a pulse of 250 kHz each 2 ms and the multiple scattered waves are captured by a coda receptor (ultrasonic transducer of central frequency 100 kHz, CTS Valpey-Fisher Corporation, model SSOO58). Both transducers were excited and amplified by a Pulser-receiver (Olympus, model 5800) used in mode through-transmission. The transmitted coda signal was first filtered in the Pulser-receiver by a high-pass filter of 100 kHz to avoid the coherent low frequency signals, then amplified by the RF-Signal Preamplifier Stanfor and finally captured by the digital oscilloscope Tektronix.

The microcracks nonlinearity are modulated by the SW pump-wave source (same ultrasound probe used in NRUS technique), which by using the function generator WaveTek sends a linear frequency sweep of 10–55 kHz each 30 ms repeated continually during the ramp-time of 16 s. During the test, the amplitude of this frequency sweep increases gradually from 10 mV to 1 V and is amplified by the power amplifier (Figure 4c). This process is performed in an intermittent fashion to compensate for any linear sound speed variations (thermal variations) [8]. The function generator trigger source is used to synchronise the process of coda wave registration through the oscilloscope and both devices are controlled by LabView software.

Before implementing the NCWI technique in the compression testing machine we carried out an initial test in order to optimise our device settings and signals processing parameters (Figure 4b). To do this, we used the same sample tested by the NRUS technique described earlier (Figure 3). The testing equipment was a Tecnotest machine suitable for bending and compression tests, with a maximum capacity of 3000 kN for compression, with a tolerance of 0.1 N. Next, we installed our ultrasonic device on the testing machine and we performed the NCWI testing on the concrete cube samples after the application of different levels of loads, from 10% to 80% of the ultimate stress level (200 to 2000 kN). We estimated the ultimate stress by testing a few concrete samples from the same batch (10 concretes samples) and we used a sample to perform the NCWI test. The ultrasound probes were placed laterally around the sample by using shear-wave couplant paste (fixed by clamps) and the load was applied gradually on its upper face (see Figure 7b). For each loading level, immediately after releasing the load, the NCWI test was performed (10 min) and the coda wave signal was registered for posterior analysis.

## 3. Results

### 3.1. Nonlinear Resonance Ultrasound Experiments

**Figure 5:**
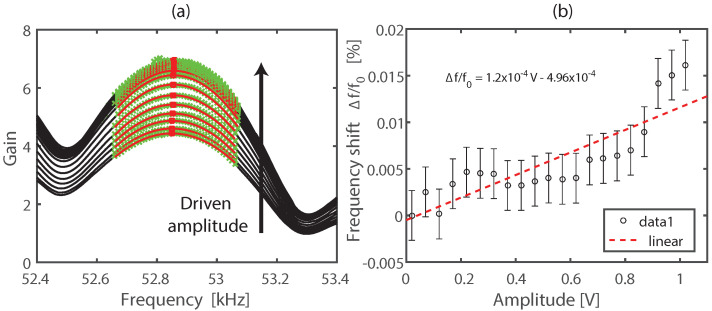
NRUS results. (**a**) Gain spectre for incremental levels of the driven amplitude. The red curve represents the second order polynomial fit around the gain peak (red colour data). (**b**) Frequency-shift in function of the driven amplitude and estimation of the nonlinear hysteretic parameter α. Error bars on (**b**) were estimated from the fitting coefficients.

Figure 5 shows the results of the application of the NRUS technique using the experimental setup shown in Figure 3 and described in Section 2.3. In Figure 5a we show the transmitted to emitted amplitude ratio (gain) in terms of the frequency for different levels of driven amplitude, which was computed in Matlab software using the peak of the Fourier spectrum. To estimate the nonlinear elastic hysteretic parameter α given by Equation (3), we compute the resonance frequency of each gain spectre by fitting a second order polynomial around its maximum (green data in Figure 5a). This fitting process (red colour curve) allows us to overcome the resolution frequency limitation of the gain spectre and then estimate the α parameter by linear fitting of the frequency-shift in terms of the driven amplitude (Figure 5b).

Despite the fact that the frequency-shift is weak, it means the concrete weakening behaviour is small, we can estimate a nonlinear elastic hysteretic parameter $\alpha \approx 1.2\times 10^{-4}$. This low nonlinear elastic behavior can be attributed to the low pump-wave amplitude achieved by our experimental system. Comparatives studies using longitudinal and SW ultrasonic probes previously calibrated will be necessary.

### 3.2. NCWI Application

**Figure 6:**
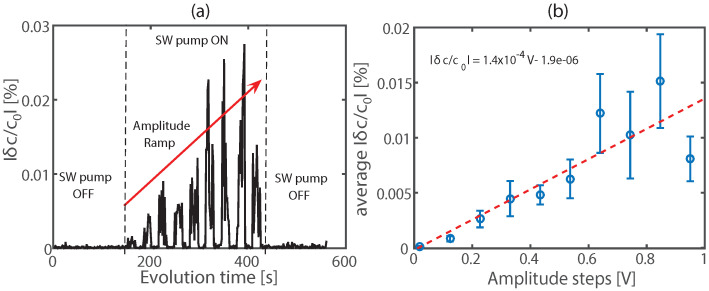
NCWI results for preliminary study. (**a**) Relative sound speed variation from CWI signal processing application. (**b**) Average sound speed variation $\delta c/c_{0}$ as a function of the SW pump-wave amplitude and estimation of the nonlinear elastic hysteretic parameter α.

Figure 6 shows the results of the application of the NCWI technique using the experimental setup described in Section 2.4. Here we used the same concrete cubic sample tested before by the NRUS technique (Section 3.1). The relative change of sound speed between two consecutive coda signals is shown in Figure 6a. The peaks observed in the central zone (SW pump is ON) correspond to the incremental variations in the sound speed produced by the SW pump-wave source (amplitude ramp). The average value of the sound speed variation $\delta c/c_{0}$ for each amplitude step of the SW pump-wave source is shown in Figure 6b. The linear fit allows us to obtain the nonlinear elastic hysteretic parameter $\alpha \approx 1.4\times 10^{-4}$, which is relatively similar to the value estimated previously by the NRUS technique (Figure 5b, Section 3.1).

**Figure 7:**
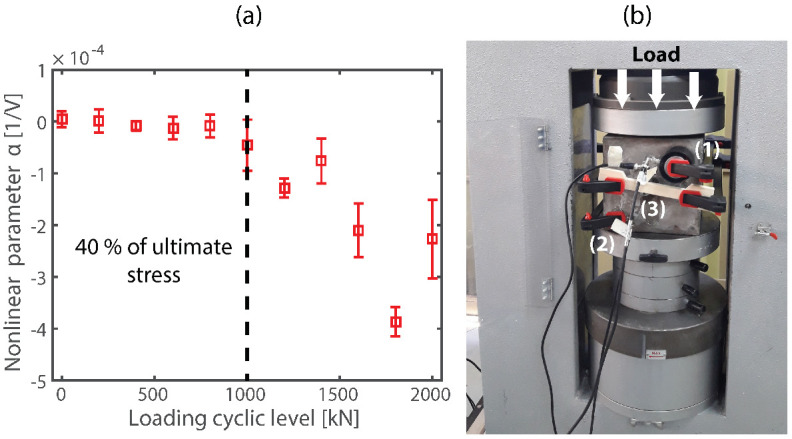
NCWI results for different loading cyclic level. (**a**) Nonlinear elastic hysteretic parameter as a function of the amplitude of the loading cyclic from 10 to 2000 KN indicating the material softening process. (**b**) Experimental setup with ultrasonic probes (1 coda emitter, 2 coda receptor and 3 SW pump-wave source) on the front face of the cubic sample coupled by shear-wave paste and fixed by clamps. For each loading cycle, the NCWI test was performed immediately after to release the load within about 10 min.

Figure 7a shows the results of the NCWI application to a concrete cubic sample after incremental cyclic loading. For each loading level, we estimate the nonlinear elastic hysteretic parameter α from the sound speed variation curve as a function of the pump-wave source amplitude (see Figure 6b). We observe that before reaching a loading level corresponding to 40% (around 1000 kN) of the ultimate stress the nonlinear parameter is almost negligible. However, after this loading level α starts to decrease gradually, showing an increment in the nonlinear hysteretic behavior where the negative value is used to show material softening (note that in Figure 6 we use absolute values). We observe an increment in dispersion data with the loading level, which may indicate problems of coupling contact between ultrasound probes induced by the concrete sample cracking (signal lost) and may also show an increment of decorrelation between coda signals. As was previously described in Section 2.4, in Figure 7b we show the experimental setup where the ultrasonic probes in the front face of the cubic sample and the load applied front the top.

Despite the purpose of this initial study was to evaluate the feasibility of using shear waves transducers as pump sources in the NCWI technique, further studies must be performed to consolidate our results, including comparative studies using longitudinal waves transducer as a pump source and obtain the stress-strain curve of the cyclic loading for our concrete samples.

## 4. Discussion

Our results show that it is technically feasible to use shear wave transducers as a pump-wave source in the NCWI technique applied to concrete specimens under laboratory conditions. Despite the fact that the nonlinear elastic hysteretic behavior is small our results are consistent with the NRUS technique and the microcracking proliferation process as a consequence of the cyclic loading. In comparison with other studies, our results are consistent with recent numerical works based on the 2D spectral element method [25,26,27,28,29] and previous experimental results. That is, softening increases as microcrack proliferation spreads as a consequence of cyclic loading. However, the lack of incorporation of nonlinear elastic behavior in this simulation technique limits the direct comparison with our results. Moreover, more exhaustive experimental studies are necessary to statistically validate our results. Furthermore, we need to perform comparative studies using longitudinal transducers as a pump-wave source, obtain the specific stress-strain curves for the cyclic loading process to characterise the mechanical properties of the concrete samples and implement methods for the visualisation of microcracks formation. In particular, both kinds of pump-wave transducers must be calibrated by measuring the level of micro-strain. After this, we can perform parametric studies to evaluate the performance of a mixed transducer and explore the effects of its relative positions. The development of these parametric experimental studies in conjunction with an appropriate simulation of nonlinear elastic behavior induced by the pump-source may help to design strategies for the selection and placement of pump-wave sources to improve the performance of the NCWI technique. These strategies may be tested in structural concrete under more realistic conditions by using embedded ultrasound transducers [17,18,19,20]. We believe that all of these studies may help to improve the performance of the NCWI technique and also to study the dynamical processes behind the microcrack growth and further large crack formation.

## 5. Conclusions

In this paper, we have studied the feasibility of using SW as a pump-wave source for concrete microcracks detection and monitoring by means of NCWI technique. The premise behind our study considers that an SW emitter can be used as a pump-wave source to induce the nonlinear elastic hysteretic behavior of microcracks oriented in directions not normally detected by a conventional longitudinal pump-wave source placed at the same position. The SW pump-wave source in conjunction with the coda probes were placed around a cubic concrete sample, which was subjected to cyclic loading. For each level of loading, the NCWI test was performed immediately after releasing the load. Our results showed that after reaching a load level of 40% of the ultimate stress, the nonlinear elastic hysteretic behaviour started to increase as a consequence of microcracks proliferation, which is consistent with previous studies. Despite the lack of exhaustive experimental studies our results support the technical feasibility of using SW transducers as a pump-wave source in the NCWI technique applied to concrete samples under laboratory conditions. However, these results represent only a first step and more experimental studies are required in conjunction with numerical simulations, to generate the basis for the design of further strategies of selection and placement of pump-sources that help to improve the performance of the NCWI technique.

## Data Availability

Not applicable.
